# Pilot Clinical Trial of Indocyanine Green Fluorescence-Augmented Colonoscopy in High Risk Patients

**DOI:** 10.1155/2016/6184842

**Published:** 2016-02-17

**Authors:** Rahul A. Sheth, Pedram Heidari, Kevin Woods, Daniel Chung, Andrew T. Chan, Umar Mahmood

**Affiliations:** ^1^Athinoula A. Martinos Center for Biomedical Imaging, Massachusetts General Hospital, Boston, MA 02114, USA; ^2^Department of Interventional Radiology, Division of Diagnostic Imaging, MD Anderson Cancer Center, Houston, TX 77030, USA; ^3^Cancer Treatment Centers of America, Southeastern Regional Medical Center, Atlanta, GA 30265, USA; ^4^Division of Gastroenterology, Massachusetts General Hospital, Boston, MA 02114, USA; ^5^Clinical and Translational Epidemiology Unit, Massachusetts General Hospital, Boston, MA 02114, USA

## Abstract

White light colonoscopy is the current gold standard for early detection and treatment of colorectal cancer, but emerging data suggest that this approach is inherently limited. Even the most experienced colonoscopists, under optimal conditions, miss at least 15–25% of adenomas. There is an unmet clinical need for an adjunctive modality to white light colonoscopy with improved lesion detection and characterization. Optical molecular imaging with exogenously administered organic fluorochromes is a burgeoning imaging modality poised to advance the capabilities of colonoscopy. In this proof-of-principle clinical trial, we investigated the ability of a custom-designed fluorescent colonoscope and indocyanine green, a clinically approved fluorescent blood pool imaging agent, to visualize polyps in high risk patients with polyposis syndromes or known distal colonic masses. We demonstrate (1) the successful performance of real-time, wide-field fluorescence endoscopy using off-the-shelf equipment, (2) the ability of this system to identify polyps as small as 1 mm, and (3) the potential for fluorescence imaging signal intensity to differentiate between neoplastic and benign polyps.

## 1. Introduction

Colorectal cancer is the second most common cause of cancer-related deaths in the United States, with 140,000 new diagnoses and 50,000 deaths per year. Effective screening and early removal of precancerous polyps are the key to the successful prevention and treatment of colorectal cancer [1]. Although there are several colorectal cancer-screening tests available, white light colonoscopy is the current gold standard for early detection and treatment.

Widespread adoption of white light colonoscopy screening appears to have had an impact on the incidence of colorectal cancer in the US, but emerging data suggest that this approach is inherently limited. First, many studies have shown that even the most experienced colonoscopists, under optimal conditions, miss at least 15–25% of adenomas [2]. Second, colonoscopy is inadequate protection against cancers in the right colon [3, 4]. Third, colonoscopy is more likely to miss morphologically “flat” adenomas, or nonpolypoid colorectal neoplasms, present in up to 10–20% of older adults. These lesions may be more likely to harbor severe dysplasia [5].

There remains a clear imperative to develop new imaging modalities that can more efficiently and objectively detect, in real time and* in situ*, neoplastic lesions throughout the gastrointestinal tract. Optical molecular imaging (OMI) with exogenously administered organic fluorochromes is a burgeoning imaging modality poised to advance the capabilities of colonoscopy. With features such as real-time, multichannel, high resolution imaging that can be performed through existing clinical endoscopes, OMI has the potential to improve colorectal cancer care through earlier disease detection [6], improved lesion visualization, and improved lesion characterization [7]. In this proof-of-principle clinical trial, we investigated the ability of a custom-designed fluorescent colonoscope and indocyanine green, a clinically approved fluorescent blood pool imaging agent, to visualize polyps in high risk patients with polyposis syndromes or known distal colonic masses.

## 2. Materials and Methods

### 2.1. Study Design

Institutional review board approval was obtained for this prospective, single-center, Health Insurance Portability and Accountability Act-compliant trial (clinicaltrials.gov identifier NCT01112514). All participants (*n* = 3) provided written informed consent. The primary end-point was detection of adenoma, cancer, or areas of dysplasia on lower endoscopy (sigmoidoscopy).

The eligibility and exclusion criteria are summarized as follows.

*Eligibility/Exclusion Criteria*

*Eligibility Criteria. *The eligibility criteria were as follows:

 High risk polyposis syndrome with planned routine endoscopic surveillance or patients with planned endoscopic evaluation of distal colonic lesions suspicious for colorectal polyps.
 Being fit for conscious sedation or monitored anesthesia care for colonoscopy (American Society of Anesthesiologists [ASA] Class I or II).
 Age ≥ 18 years.
 ECOG performance status ≤ 2.

*Exclusion Criteria. *The exclusion criteria were as follows:

 ASA class III, IV, or V.
 Documented allergy to iodine, iodine-containing compounds, or ICG.
 Documented allergy to sulfur containing products.
 Pregnancy.

Participants were identified through outpatient clinics at our institution's high risk cancer genetics clinic or Digestive Health Care Center. Eligible participants were patients with high risk polyposis syndromes with planned lower endoscopic procedures for screening or surveillance for polyps or dysplasia, or patients who had planned lower endoscopies to evaluate a distal colorectal mass visualized on imaging. Participants were not offered endoscopy for the sole purpose of participation in this trial. Participation in this study did not interfere with other planned therapeutic interventions. Follow-up consisted of phone call interviews on postoperative day (POD) 2 and POD 8.

### 2.2. Fluorescence Endoscopy System

**Figure 1:**
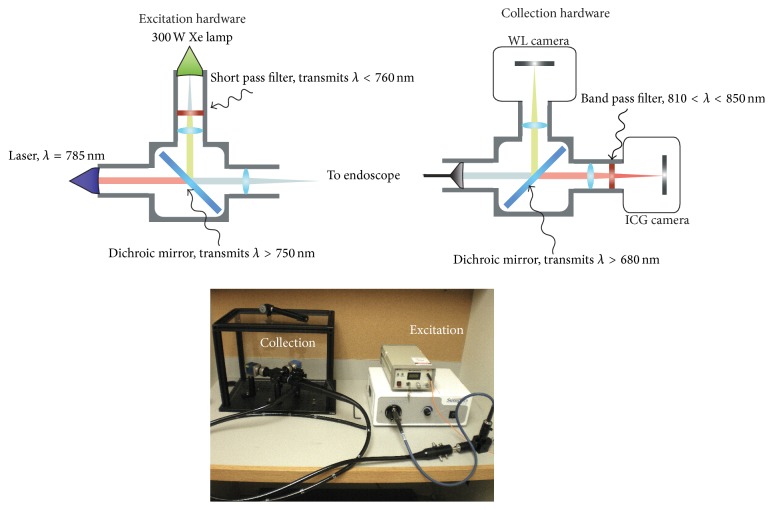
Schematic and photograph of dual channel clinical fluorescence endoscope.

A fluorescence endoscopy system was constructed in a manner analogous to previously designed devices and incorporated several technologies including quantitative real-time fluorescence imaging developed by our group [8–12] (Figure 1). The endoscope used in this trial was a clinically approved fiber optic sigmoidoscope (Pentax). This endoscope was adapted so that the illumination input was coupled to a custom-designed optical apparatus that allows for the illumination of standard white light as well as near infrared (NIR) light for fluorescence imaging. The NIR laser optimized for ICG excitation was a 785 nm 450 mW fiber-coupled laser (Edmund Optics, Barrington, NJ, USA). The white light illumination was provided by a metal halide 150 W lamp (Edmund Optics, Barrington, NJ, USA). The optical output for the sigmoidoscope was attached via a custom-designed eyepiece adapter to an optical imaging system that allowed for the imaging of standard white light as well as ICG fluorescence. Although this device had custom-designed light sources, only clinically approved equipment (i.e., the fiber optic sigmoidoscope) was introduced into the patient.

### 2.3. Fluorescence Endoscopy Procedure

The study endoscopy was performed by board-certified gastroenterologists in tandem with the participant's already planned clinical lower endoscopy. Participants underwent the study fluorescence lower sigmoidoscopy with white light and NIR using the study endoscope with ICG administration. After this examination was completed, the participant underwent a routine exam with a clinical videoendoscope per usual care. This routine clinical endoscope was used to reinspect the distal colon and rectum previously examined by the experimental endoscope to ensure that no lesions were missed on the experimental examination.

ICG was prepared for intravenous injection by the institutional research pharmacy. An initial intravenous injection of ICG at 8 mg was delivered during NIR sigmoidoscopy. Follow-up doses between 1 mg and 8 mg were given over the course of the endoscopy, not to exceed a total dose of 24 mg. The maximum amount of ICG to be administered during the procedure was 24 mg (3 doses of 8 mg each). If a clear view was obtained with any dose less than 24 mg, more ICG was not given. The minimum amount of ICG was not set and was individualized according to the visual clarity in each participant.

To best isolate the additional benefit of ICG + NIR over white light, we used the same endoscope to deliver both NIR and white light to ensure that any differences in the number of lesions detected were due to the ICG and NIR and not due to different optical properties of the experimental endoscope compared with the clinical endoscope. The experimental endoscopy was performed first so that the endoscopist was not biased by knowledge of the findings from the clinical endoscopy. By performing the clinical endoscopy after the experiment examination, this also allowed the clinical endoscopy to be a “clearing endoscopy” to ensure that the participant had optimal removal of all neoplastic lesions according to standard of care for a surveillance examination.

The endoscopist first used the experimental endoscope with white light only and advanced the endoscope into the distal sigmoid colon or, where relevant, the surgical anastomosis for patients who had colectomies. In the white light phase, for any lesions suspicious for neoplasm, the endoscopist photodocumented the lesions, classified morphology using previously defined classification schemes (mucosal lesion, flat elevation, or depression, or no mucosal abnormality), and estimated the size of the lesion with comparison to biopsy forceps. After completing the white light diagnostic examination, the participant received 8 mg dose of ICG intravenously. The endoscopist then conducted the second diagnostic phase by reexamining using fluorescence following the same protocol. In this phase, photodocumentation of lesions also occurred using the NIR camera. * *Using the modality that initially identified the lesion, the endoscopist then performed biopsy/excision of the lesion using standard biopsy forceps or loop snares.

After completion of the study endoscopy, the endoscopist performed a routine clinical endoscopy as per usual clinical protocol. The distal colon and rectum previously inspected using the experimental endoscope were reinspected using a standard clinical videoendoscope. Any additional lesions using the standard clinical endoscope were biopsied and removed as per routine clinical care.

### 2.4. Image Analysis

Analysis of the fluorescence data was performed by drawing regions of interest (ROIs) within the polyp using a standard software package and measuring the mean pixel intensity (ImageJ; National Institutes of Health, Bethesda, MD). Target-to-background ratios (TBRs) were calculated by drawing ROIs within adjacent normal colonic mucosa on the same image and dividing the polyp's mean fluorescence intensity with mucosal fluorescence intensity.

## 3. Results

### 3.1. Patient Population

The demographics for the trial participants are summarized in Table 1. A total of 3 participants were enrolled in the study. All patients had a prior history of neoplastic polyps, and two patients had previously been diagnosed with familial adenomatous polyposis (FAP) syndrome. All patients underwent experimental ICG fluorescence endoscopy followed by conventional endoscopy. Biopsies of polyps were performed either during initial fluorescence endoscopy, during conventional biopsy, or both. Polyps as small as 1 mm were well visualized with the experimental endoscope; the median polyp size for all participants was 3.5 mm. A total of 3 nonneoplastic polyps and 3 neoplastic adenomas (tubular adenomas) were visualized by endoscopy and biopsied. Using a TBR cutoff of 1.2, the true positive, true negative, false positive, and false negative values for ICG enhancement are presented in Table 2. Using these values, sensitivity was 0.67; specificity was 1.0; positive predictive value was 1.0; and negative predictive value was 0.75. No major adverse events were identified in this study.

### 3.2. Patient 1

**Figure 2:**
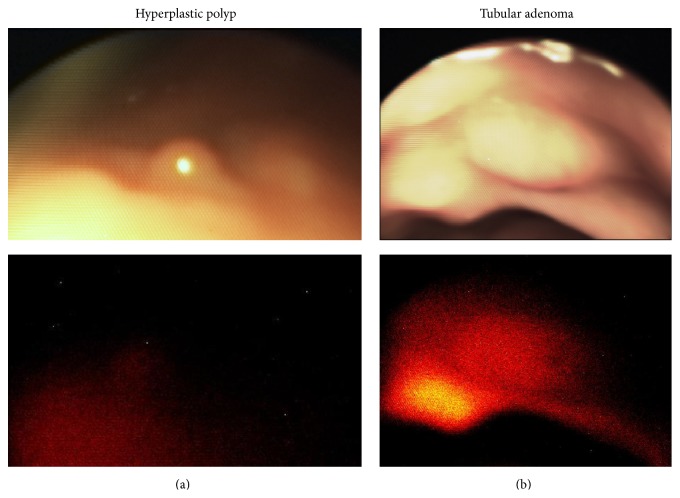
Patient 1 fluorescence endoscopy. A benign polyp (hyperplastic, left) demonstrates no substantial ICG uptake over background (TBR 1.0), while a neoplastic one (tubular adenoma, right) does (TBR 1.7).

Patient 1 was a 51-year-old male with a history of chronic alcohol abuse who initially presented with chronic anemia. He underwent conventional colonoscopy one year prior to trial enrollment and was found to have a flat rectal adenoma. This lesion underwent serial partial piecemeal removal with 2 subsequent endoscopies. The patient was enrolled in the study for his fourth endoscopy to debulk the lesion. During experimental endoscopy, the previously identified adenoma was seen 15 cm from the anal verge and measured approximately 18 mm (Figure 2). The adenoma demonstrated ICG uptake with a TBR of 1.8 ± 0.08. Partial removal was performed during both experimental and subsequent conventional endoscopy. In addition, a second polyp measuring 3 mm was seen 20 cm from the anal verge on both fluorescence and conventional endoscopy. There was no increased ICG uptake relative to background (TBR 1.0 ± 0.08). This lesion was biopsied through the experimental endoscope and found to be a hyperplastic polyp. The patient tolerated the trial procedure without complication and demonstrated no adverse events on follow-up phone calls on POD 2 and 8.

### 3.3. Patient 2

**Figure 3:**
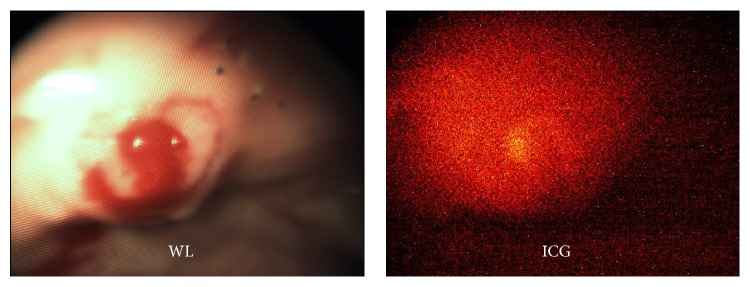
Patient 2 fluorescence endoscopy. A 10 mm tubular adenoma demonstrates slightly greater ICG uptake over background (TBR 1.21).

**Figure 4:**
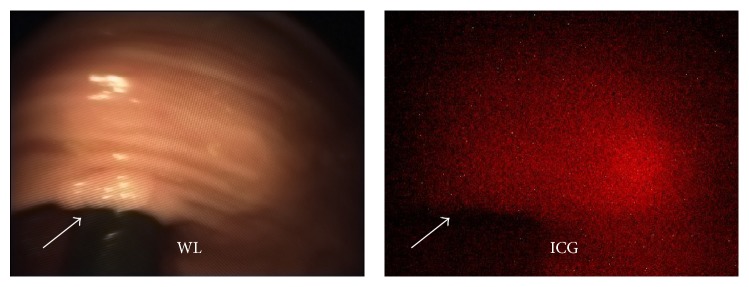
Patient 2 fluorescence endoscopy. A tubular adenoma is indistinguishable from adjacent normal colonic mucosa on ICG imaging, representing a false negative.

Patient 2 was a 49-year-old male with a history of FAP and prior subtotal colectomy and small bowel resections. He was enrolled in the trial at the time of a scheduled high risk surveillance endoscopy. A total of 4 polyps were identified in the rectum on both experimental and conventional endoscopy and removed in entirety. Specifically, two 1 mm sessile polyps were seen at 2 cm and 4 cm from the anal verge. Neither of these lesions demonstrated substantial ICG uptake above background (TBRs 1.08 ± 0.08 and 1.06 ± 0.08) and were found containing normal colonic mucosa on pathology. A third polyp was seen 10 cm from the anal verge measuring 10 mm; this polyp exhibited ICG uptake slightly greater than background (TBR 1.21 ± 0.08) and was found to be a tubular adenoma on pathology (Figure 3). A fourth polyp at 15 cm beyond the anal verge that measured 4 mm was seen and found to represent a tubular adenoma. However, ICG uptake within this adenoma was not above background (TBR 1.0 ± 0.08), consistent with a false negative (Figure 4). The patient complained of emesis following a meal on POD 2 but was asymptomatic on POD 8.

### 3.4. Patient 3

Patient 3 was a 51-year-old female with FAP who had undergone multiple prior colonoscopies to remove neoplastic polyps, followed by total colectomy with ileoanal anastomosis. On initial experimental endoscopy, no polyps were seen. However, subsequent conventional endoscopy revealed two sessile polyps (2 mm each) at the rectal cuff. Given this finding, repeat experimental endoscopy was performed. However, due to relative limitations of the experimental endoscope compared to the conventional endoscope including decreased flexibility, the known polyps were again not well visualized. Both polyps on histology were found to be tubulovillous adenomas. The patient complained of emesis following a meal on POD2 and headache on POD 8.

## 4. Discussion

**Figure 5:**
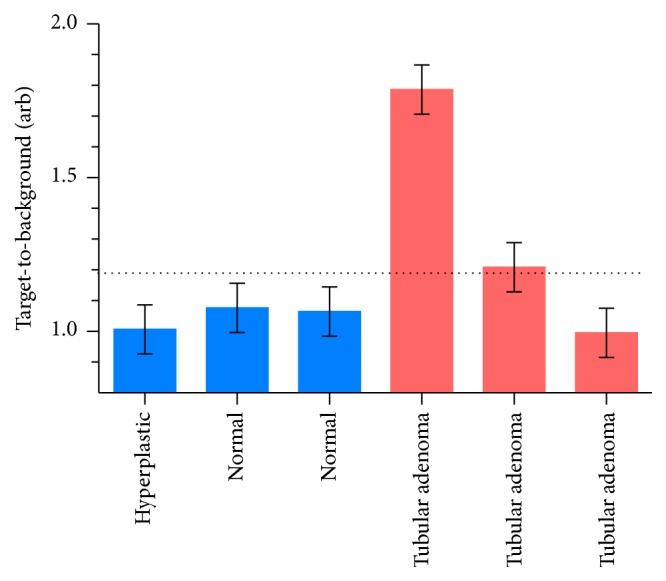
ICG TBRs for all polyps identified and biopsied in the clinical trial. Horizontal dotted line = TBR cutoff of 1.2.

We have demonstrated the feasibility of fluorescence imaging as a complement to conventional white light colonoscopy. We applied a custom-designed dual functionality colonoscope and performed simultaneous ICG-enhanced fluorescence and standard white light imaging in a clinical trial setting. We have shown (1) the successful performance of real-time, wide-field fluorescence endoscopy using off-the-shelf equipment, (2) the ability of this system to identify polyps as small as 1 mm, and (3) the potential for fluorescence imaging signal intensity to differentiate between neoplastic and benign polyps (Figure 5).

White light colonoscopy is the standard of care surveillance and early intervention tool for colorectal cancer. Colonoscopy relies on the ability of physicians to visually identify and resect any lesions that are potentially adenomas. Because nonneoplastic polyps (e.g. hyperplastic polyps) can be morphologically indistinguishable from adenomas, physicians typically remove all polyps during a procedure and send them for histopathological correlation. Beyond the inability of conventional colonoscopy to characterize polyps* in situ*, tandem colonoscopy studies have estimated the rate of “missed” adenomas at 15–25% [2]. Additionally, current colonoscopic surveillance for dysplasia in the setting of ulcerative colitis or Crohn's disease poses a unique set of challenges. Patients' risk of cancer rises dramatically with increasing extent, severity, and duration of inflammation [13]. Although neoplasia can be detected in colitis patients as a dysplasia-associated lesion or mass, these lesions can be difficult to distinguish morphologically from benign inflammatory polyps and dysplastic mucosa that appears endoscopically unremarkable. Thus, the current standard of multiple random biopsies over the entire colorectum during a colonoscopy is not only insensitive, but resource-intensive, time- consuming, and not widely adopted [14]. And the sensitivity for cancer is severely limited since it relies on fortuitous sampling of dysplastic mucosa. Indeed, as many as 50% of ulcerative colitis patients that develop cancer over 30 years had a previously unremarkable colonoscopy [13].

OMI with fluorescent probes is well-suited to address the limitations of conventional colonoscopy. As a real-time imaging modality that is compatible with current clinical devices, OMI can augment white light imaging by highlighting areas of abnormality. A vast array of molecularly targeted optical imaging agents has been developed for preclinical use [7], targeting pathways known to be dysregulated in colorectal cancer. Examples include matrix metalloproteinases [15], caspases [16], vascular endothelial growth factor [17], and epidermal growth factor receptor [18]; a clinical trial of an enzyme-activatable OMI agent is also underway (NCT01626066). Complementing the development of OMI probes, sophisticated fluorescence imaging systems have been developed, including a clinically approved confocal microendoscope [18–23]. This device has been applied to the detection of a topically applied OMI agent during clinical colonoscopies [19]. Confocal imaging systems maximize spatial resolution at the expense of field-of-view. We have shown that wide-field-of-view fluorescence colonoscopy using targeted OMI agents is possible in orthotopic mouse models [9, 24]; this trial represents the clinical translation of such prior work.

The fluorescent probe used in this clinical trial, ICG, has been clinically approved for decades. ICG binds to serum albumin, prolonging its intravascular half-life and allowing it to function as a blood pool imaging agent. As such, ICG does not exhibit any enhanced adenoma specificity other than the relative hypervascularity of these lesions compared to colonic mucosa. Nonetheless, we were able to demonstrate the ability to visualize a degree of adenoma specificity based on fluorescence imaging. While fluorescence endoscopy was limited to the sigmoid colon in this study, the technology and equipment can be readily applied for full colonic evaluation; additional boluses of ICG would substantially raise the toxicity profile. Though we encountered a false negative based upon ICG signal intensity and we observed the limitations of the experimental endoscope in patients with challenging postoperative anatomy, this case series represents a successful proof-of-concept for combining OMI with colonoscopy. Ultimately, to illustrate improved adenoma detection and polyp characterization over standard of care colonoscopy, similar trials that utilize truly molecularly targeted fluorescent agents will be necessary.

## Figures and Tables

**Table 1:** Demographics of trial participants. ^*∗*^The polyps seen in Participant 3 were only visualized on conventional endoscopy and not on experimental endoscopy.

Participant	Gender	Age	Medical history	Prior surgery	# polyps	
					Nonneoplastic	Neoplastic
1	M	51	EtOH abuse	None	1 (3 mm)	1 (18 mm)
2	M	49	FAP	Subtotal colectomy	2 (1 mm, 1 mm)	2 (4 mm, 10 mm)
3	F	51	FAP	Total colectomy with ileoanal anastomosis	0	2 (2 mm, 2 mm)^*∗*^

**Table 2:** Evaluation of ICG uptake as a biomarker for polyp classification. A TBR > 1.2 as the cutoff value for neoplasia was used.

ICG +	Neoplastic	
	Yes	No
Yes	2	0
No	1	3
